# Assessment of Rural-Urban and Geospatial Differences in Perceived Handgun Access and Reported Suicidality Among Youth in Colorado

**DOI:** 10.1001/jamanetworkopen.2021.27816

**Published:** 2021-10-08

**Authors:** Talia L. Spark, Erin Wright-Kelly, Ming Ma, Katherine A. James, Colleen E. Reid, Ashley Brooks-Russell

**Affiliations:** 1 Rocky Mountain Mental Illness Research, Education, and Clinical Care, Department of Veteran Affairs, Aurora, Colorado; 2 Physical Medicine and Rehabilitation, School of Medicine, University of Colorado Anschutz Medical Campus, Aurora; 3 Injury and Violence Prevention Center, University of Colorado Anschutz Medical Campus, Aurora; 4 Department of Community and Behavioral Health, Colorado School of Public Health, University of Colorado Anschutz Medical Campus, Aurora; 5 Department of Environmental and Occupational Health, Colorado School of Public Health, University of Colorado Anschutz Medical Campus, Aurora; 6 Geography Department, University of Colorado, Boulder

## Abstract

**Importance:**

Suicide is the second leading cause of death in adolescents, with firearms the most common method, especially in rural communities. Identifying where to target lethal means safety interventions could better leverage limited resources.

**Objectives:**

To understand the associations of rurality, school-level prevalence of easy handgun access, and suicidality measures in Colorado youth, to explore spatial distribution of school-level measures, and to identify communities with high prevalence of both easy handgun access and suicidality.

**Design, Setting, and Participants:**

This cross-sectional study used data from the 2019 Healthy Kids Colorado Survey, an anonymous cross-sectional school-based survey conducted at 256 participating Colorado high schools. Participants included students from schools recruited for statewide population-based estimates and additional schools opting in. Data were analyzed from November 9, 2020, to March 13, 2021.

**Exposures:**

Urban-centric locale according to a 7-level continuum. Geocoded location of schools was used for spatial analysis.

**Main Outcomes and Measures:**

The main outcomes were weighted prevalence for easy handgun access and 4 measures of mental health and suicidality in the previous year (ie, feeling sad for 2 weeks and considering suicide, planning suicide attempt, or attempting suicide in the past year).

**Results:**

A total of 59 556 students (49.7% [95% CI, 49.3%-50.1%] male and 50.3% [95% CI, 49.9%-50.7%] female; 53.9% [95% CI, 53.5%-54.3%] in 9th and 10th grade; 36.4% [95% CI, 36.0%-36.8%] Hispanic and 50.8% [95% CI, 50.4%-51.2%] non-Hispanic White) from 256 schools participated. Most schools were rural or in small towns (56.8% [95% CI, 50.7%-62.9%]), while more students participated from urban and suburban schools (57.8% [95% CI, 57.6%-58.0%]). Prevalence of perceived easy access to handguns increased with increasing rurality, with 36.2% (95% CI, 35.2%-37.1%) of students in rural (remote) schools reporting easy access, compared with 18.2% (95% CI, 17.3%-19.1%) for city (large) schools. The spatial distribution of easy handgun access and suicidality measures had minimal overlap, but there was correlation at school-level between easy handgun access and considering suicide (ρ = 0.203 [95% CI, 0.0748-0.331]), planning suicide (ρ = 0.300 [95% CI, 0.173-0.427]), and attempting suicide (ρ = 0.218 [95% CI, 0.0869-0.350) in the previous year. The highest quartile for prevalence of both perceived easy access to handguns and planning suicide in the previous year included 21 schools (81.0% [95% CI, 64.0%-97.9%] rural [remote] or rural [distant]).

**Conclusions and Relevance:**

These findings suggest that rural-remote communities in Colorado may benefit most from interventions focused on limiting youth access to handguns when youth are in crisis, with some communities at especially high risk. Spatially referenced data may improve targeting interventions to where they are needed most.

## Key Points

**Question:**

Are rurality and geography associated with access to handguns and suicidality in Colorado youth?

**Findings:**

In this cross-sectional study including 59 556 Colorado high school students, rurality and geography correlated with reported prevalence of easy handgun access and suicidality.

**Meaning:**

These findings suggest that incorporating geographic information, beyond measures of rurality, could inform where to focus youth firearm safety efforts to prevent suicide.

## Teaser

This cross-sectional study examines the associations of rurality, self-reported ease of handgun access, and suicidality measures among high school students in Colorado.

## Introduction

Suicide is the second leading cause of death for youth aged 15 to 19 years in the United States,^1^ with rates increasing 76% from 2007 to 2017.^2^ Firearms are the leading method, accounting for 51% of all youth suicide deaths.^3^ Access to firearms is associated with increased suicide rates.^4,5,6,7,8,9,10^ Although simply owning a firearm is not associated with increased suicidality,^11,12^ the outcome is more likely lethal when individuals in crisis have access to firearms.^13,14^ While the best practice is to keep firearms out of the home, households with children opting to own firearms should limit access by storing all firearms locked, unloaded, with ammunition locked and stored separately. However, a 2018 study reported that only 30% of households with children and firearms reported all firearms were stored locked and unloaded.^15^

Firearm availability and youth firearm suicide risk vary geographically within the US.^15,16,17,18^ Rural youth are at 2-fold increased risk of dying by firearm suicide compared with their urban counterparts.^19^ Moreover, individuals in rural communities are more than 2-fold more likely than those living in urban communities to own firearms.^20^ To our knowledge, no surveillance systems track youth firearm access nationally or at smaller scales.^21,22^

To address this limitation, in 2019, the Healthy Kids Colorado Survey (HKCS), a state-wide biennial cross-sectional survey of middle and high school students, began asking high school students whether they could easily get a handgun if they wanted one. Initial findings indicated that nearly 1 in 5 high school students reported having easy access to handguns.^23^ However, little is known about youth firearm access across geographic contexts. Identifying and understanding areas where combined high handgun access and suicidality overlap could inform targeted youth-focused interventions to prevent youth firearm access for those at risk of suicide.^24^

The objectives of this study were to explore the associations of rurality, school-level prevalence of easy handgun access, and suicidality measures in Colorado youth, to examine the spatial distribution of school-level easy handgun access and suicidality measures, and to identify schools where easy handgun access and measures of suicidality were both high. We hypothesized we could identify areas that might benefit most from targeted interventions in collaboration with local school districts and communities.

## Methods

This cross-sectional study was approved by the Colorado Multiple Institutional Review Board. Participation was voluntary and approved by parents; therefore, participation was considered consent. No identifying information was collected from students. This study is reported following the Strengthening the Reporting of Observational Studies in Epidemiology (STROBE) reporting guideline for cross-sectional studies.

### Participants and Procedures

Colorado public high school students received the HKCS survey between August and December of 2019. A portion of survey questions came from the Centers for Disease Control and Prevention’s Youth Risk Behavior Surveillance System, and administration was consistent with Centers for Disease Control and Prevention methods.^25^ Although the HKCS used a 2-stage stratified cluster sampling design to produce state estimates, any public middle or high school not selected in the state sample could opt-in and have students complete the questionnaire. Data from opt-in schools were included in this study to increase sample size. The anonymous survey was self-administered in classrooms during a regular class period using either a machine-scannable paper booklet or online, as determined by the school.

### Measures

We used a cross-sectional spatial analysis to explore co-occurrence of different measures across schools and geographies, with no measure treated as exposure or outcome. The measures of interest were perceived handgun access, suicidality, and urban-centric locale.

#### Perceived Handgun Access

The 2019 HKCS administration added the question: “If you wanted to get a handgun, how easy would it be for you to get one?” (response options: “Very hard,” “Sort of hard,” “Sort of easy,” and “Very easy”).^26^ For ease of presentation and analysis, we combined “Sort of easy” and “Very easy” into easy access and “Sort of hard” and “Very hard” into hard access.

#### Suicidality

Students were asked about their mental health with 4 Youth Risk Behavior Surveillance System questions: “During the past 12 months, did you ever feel so sad or hopeless almost every day for two weeks or more in a row that you stopped doing some usual activities?”; “During the past 12 months, did you ever seriously consider attempting suicide?”; “During the past 12 months did you ever make a plan about how you would attempt suicide?” (all with the response options of yes and no); and “During the past 12 months, how many times did you actually attempt suicide?” with response options of 0 times, 1 time, 2 or 3 times, 4 or 5 times, and 6 or more times, which were collapsed into 1 time or more vs 0 times.

#### Urban-Centric Locale

The National Center for Education Statistics Education Demographic and Geographic Estimates program assigned 12 urban-centric locale codes covering 4 types of areas (city, suburb, town, and rural) with 3 subcategories each to every school in the US (eTable 1 in the Supplement).^27^ Each school’s urban-centric locale assignment was based on actual location, using US Census Bureau’s definition of urbanized areas and urban clusters. We collapsed the 12 categories into 7 owing to small numbers of schools in some categories, keeping schools similar in size and proximity to cities together. This resulted in the following categories: city (large), city (midsize/small), suburb (large/midsize/small) and town (fringe), town (distant/remote), rural (distant), rural (fringe), and rural (remote). School location was obtained from the Colorado Department of Education.^28^

### Statistical Analysis

Descriptive and spatial analyses across urban-centric locale categories were completed to explore statistical and spatial differences in prevalence for key outcome and risk factor variables. Spatial analysis and visualization were used to identify communities at potentially higher risk for youth suicide.

#### Descriptive Analysis

Students were weighted by school enrollment in each participating high school. The weights were constructed to account for sampling design (clustered by classroom), student nonresponse, and discrepancies in grade, sex, and race and ethnicity between the sample and school enrollment. Race and ethnicity were self-reported. In this study, race and ethnicity were used to weight results, describe survey participants, and demonstrate differences across participants in different urban-centric locale. Initial survey analyses were conducted in SAS statistical software version 9.4 (SAS Institute) using SURVEY procedures.

Weighted prevalence and 95% CIs of easy gun access and suicidality (ie, sad for 2 weeks or considering, planning, or attempting suicide in previous year) measures were stratified by urban-centric locale categories. Significant differences between prevalence measures in city (large) schools and schools in other locales were determined using logistic regression (ie, city [large] was the reference group) taking survey design into account. Spearman rank correlation coefficient measured the correlation of easy access to handgun and suicidality measures.^29^

#### Spatial Analysis

We created a map to show the spatial distribution of urban-centric locale for each high school. To preserve school confidentiality, geomasking was used to randomly move the location of each school within a maximum distance, so schools appear in their region but not exact location.^30^

We used global Moran *I* to statistically test spatial dependence, or whether schools closer together were more likely to have similar values for each variable.^31^ Moran *I* is a measure of autocorrelation within 1 variable based on geographic neighbors, in which higher Moran *I* values indicate that a given value is more likely to be spatially proximate to other values similar to itself. A value of 0 indicates a random patterning of that variable throughout space. For these tests, we used distance-based weight matrices to define neighbors such that each school was assigned at least 1 nearest neighbor, using 42.0 miles as the threshold.^32^ To calculate significance, we used 1000 Monte-Carlo simulations to estimate *P* values. For variables with significant global Moran *I* (*P* = .05), we calculated local Moran *I* for each school to identify spatial clusters of high values and low values.^33^ We sensitivity tested the spatial autocorrelation tests by additionally evaluating Moran *I* using the minimum distance for all schools to have at least 2 neighbors (44.0 miles). Global and local Moran *I* were calculated using spdep package in R statistical software version 4.0.2 (R Project for Statistical Computing).

Using empirical Bayesian kriging, we created maps to show the spatial smoothed prevalence of each study variable.^34^ This technique was used to protect confidentiality of participating schools while visualizing spatial variation.

Schools with a prevalence in the top quartile for both easy handgun access and planning suicide were identified. Counties with at least 1 school meeting these criteria were considered to demonstrate areas with potentially higher risk of youth firearm suicide. Mapping was conducted using ArcGIS Pro version 4.6.0 (Esri).

*P* values for Moran *I* were 1-sided, other *P* values were 2-sided, and statistical significance was set at *P* = .05 for all tests. Data were analyzed from November 9, 2020 to March 13, 2021.

## Results

### Sample Characteristics

Colorado has 646 high schools (grades 9-12). Of these, 262 participated in the HKCS in 2019. We used 256 schools in this analysis (5 schools were excluded because they did not have an urban-centric locale designation, 1 school was excluded because the school removed the gun access question). The sample included 59 556 students (49.7% [95% CI, 49.3%-50.1%] male and 50.3% [95% CI, 49.9%-50.7%] female; 53.9% [95% CI, 53.5%-54.3%] in 9th and 10th grade; 36.4% [95% CI, 36.0%-36.8%] Hispanic and 50.8% [95% CI, 50.4%-51.2%] non-Hispanic White) (eTable 2 in the Supplement).

### Descriptive Analysis

**Figure 1.**
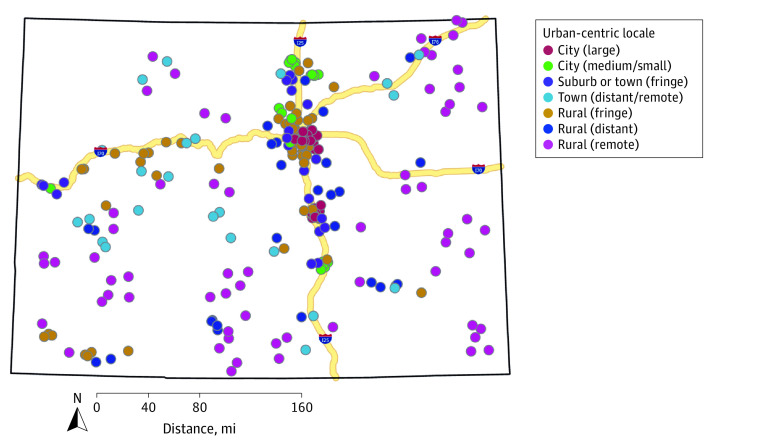
Map of National Center for Education Statistics Urban Centric Locale Designation for Each School Participating in 2019 Healthy Kids Colorado Survey Geomasking was used to protect school confidentiality. Points on map are in similar regions as actual school, but do not represent the actual location of schools.

Figure 1 presents a map of participating schools by urban-centric locale. Most schools were rural (distant, fringe, or remote) (56.8% [95% CI, 50.7%-62.9%]) with rural (remote) being the largest category. However, rural (remote) schools only represented 2.9% (95% CI, 2.9%-3.0%) of students across the state with weighted survey results (Table 1). Easy access to handguns increased in a nearly stepwise fashion, from 18.2% (95% CI, 17.3%-19.1%) in city (large) schools to 36.2% (95% CI, 35.2%-37.1%) in rural (remote) high schools, an approximately 2-fold difference (Table 1). Both considering and planning suicide in the previous year were also highest in rural (distant) and rural (remote) schools compared with city (large) schools (considering suicide: 18.6% [95% CI, 17.6%-19.4%] of students in rural [remote] schools vs 16.5% [95% CI, 15.8-17.2%] of students in city [large] schools; planning suicide: 15.6% [95% CI, 14.9%-16.3] of students in rural [remote] schools vs 12.8% [95% CI, 12.1%-13.5%] of students in city [large] schools) (Table 1).

**Table 1.** Characteristics of Schools by Locale Category, Including Weighted Prevalence For Study Measures

Urban-centric locale designation^a^	Participating schools, No. (%)	Students, No.		Estimated prevalence, % (95% CI)				
		Unweighted	Weighted^b^	Easy handgun access	Sad for 2 wk in last year	Consider suicide in last year	Plan suicide in last year	Attempt suicide in last year
Total	256	59 556	187 225	20.2 (19.8-20.6)	35.3 (34.8-35.8)	17.6 (17.2-18.0)	13.7 (13.3-14.0)	7.8 (7.5-8.0)
City (large)	36 (14.1)	10 422	39 301	18.2 (17.3-19.1)	35.8 (34.7-36.9)	16.5 (15.8-17.2)	12.8 (12.1-13.5)	8.4 (7.8-9.0)
City (midsize/small)	23 (9.0)	7379	30 027	17.7 (16.6-18.7)	35.2 (33.9-36.6)	18.4 (17.6-19.3)^c^	13.9 (13.1-14.8)^c^	7.8 (7.1-8.5)
Suburb (large/midsize/small) and town (fringe)	56 (21.9)	18 156	75 024	18.7 (17.9-19.4)	35.2 (34.3-36.0)	18.0 (17.3-18.7)^c^	13.6 (13.0-14.2)	7.2 (6.8-7.7)^c^
Town (distant/remote)	38 (14.8)	10 561	17 051	26.2 (25.5-26.9)^c^	36.0 (35.1-36.9)	17.3 (16.6-18.0)	14.3 (13.7-14.9)^c^	7.8 (7.4-8.2)
Rural (fringe)	21 (8.2)	5538	15 838	20.8 (19.6-22.0)^c^	33.9 (32.5-35.3)^c^	16.6 (15.4-17.9)	13.8 (12.7-14.9)	7.6 (6.7-8.4)
Rural (distant)	20 (7.8)	3138	4513	32.2 (30.9-33.4)^c^	37.0 (35.7-38.2)	18.6 (17.6-19.7)^c^	14.7 (13.8-15.6)^c^	9.3 (8.6-10.0)
Rural (remote)	62 (24.2)	4362	5471	36.2 (35.2-37.1)^c^	34.4 (33.5-35.4)	18.6 (17.8-19.4)^c^	15.6 (14.9-16.3)^c^	9.1 (8.5-9.7)

^a^ Urban-centric locale designation provided by National Center for Education Statistics defined based on proximity to principal cities and urbanized areas. The 12 categories were collapsed into 7 to maximize the number of schools and comparability within each group.

^b^ Weighted to represent all schools in each urban-centric locale category. Weights accounted for sampling design, student nonresponse, and discrepancies in grade, sex, and race and ethnicity between the sample and school enrollment.

^c^ Indicates significantly different than the city (large) group at *P* = .05 level. Significance tested using logistic regression.

Spearman rank correlation between school-level weighted prevalence of easy gun access and suicidality variables identified significant correlation between school-level prevalence of easy handgun access and considering suicide (ρ = 0.203 [95% CI, 0.0748 to 0.331]; *P* = .001), planning suicide (ρ = 0.300 [95% CI, 0.173 to 0.427]; *P* < .001), and attempting suicide (ρ = 0.218 [95% CI, 0.0869 to 0.350]; *P* < .001) in the previous year. Significant correlation also existed between measures of considering, planning, or attempting suicide (considering and planning: ρ = 0.808 [95% CI, 0.745-0.871]; *P* < .001; considering and attempting: ρ = 0.717 [95% CI, 0.643-0.792]; *P* < .001; planning and attempting: ρ = 0.686 [95% CI, 0.600-0.772]; *P* < .001). We observed no significant correlation between easy handgun access and prevalence of sadness for 2 weeks (ρ = 0.119 [95% CI, −0.122 to 0.250]; *P* = .06).

### Spatial Analysis

All variables, except considering suicide, showed significant spatial autocorrelation (Table 2), suggesting spatial dependence of measures. The highest global Moran *I* values were observed for weighted prevalence of easy handgun access (*I* = 0.332; *P* = .001) and planning suicide had (*I* = 0.107; *P* = .001).

**Table 2.** Global Moran *I* Statistic for Main Study Variables

Variable	Primary analysis^a^		Secondary analysis^b^	
	Moran *I*	*P* value^c^	Moran *I*	*P* value^c^
Easy handgun access	0.332	.001	0.326	.001
In last year				
Sad for 2 wk	0.0439	.03	0.0744	.005
Consider suicide	0.0116	.21	0.0288	.06
Plan suicide	0.107	.001	0.117	.001
Attempt suicide	0.0544	.02	0.065	.01

^a^ Analysis used distance-based weight matrix using the minimum-maximum criterion, ensuring each school had at least 1 neighbor.

^b^ Secondary analysis used distance-based weight matrix that ensured each school had at least 2 neighbors.

^c^ *P* value calculated using Monte-Carlo simulation with 1000 permutations. Significant positive Moran *I* indicates significant spatial autocorrelation, or that that specific measure or variable is spatially clustered with higher values near higher values and lower values near lower values.

**Figure 2.**
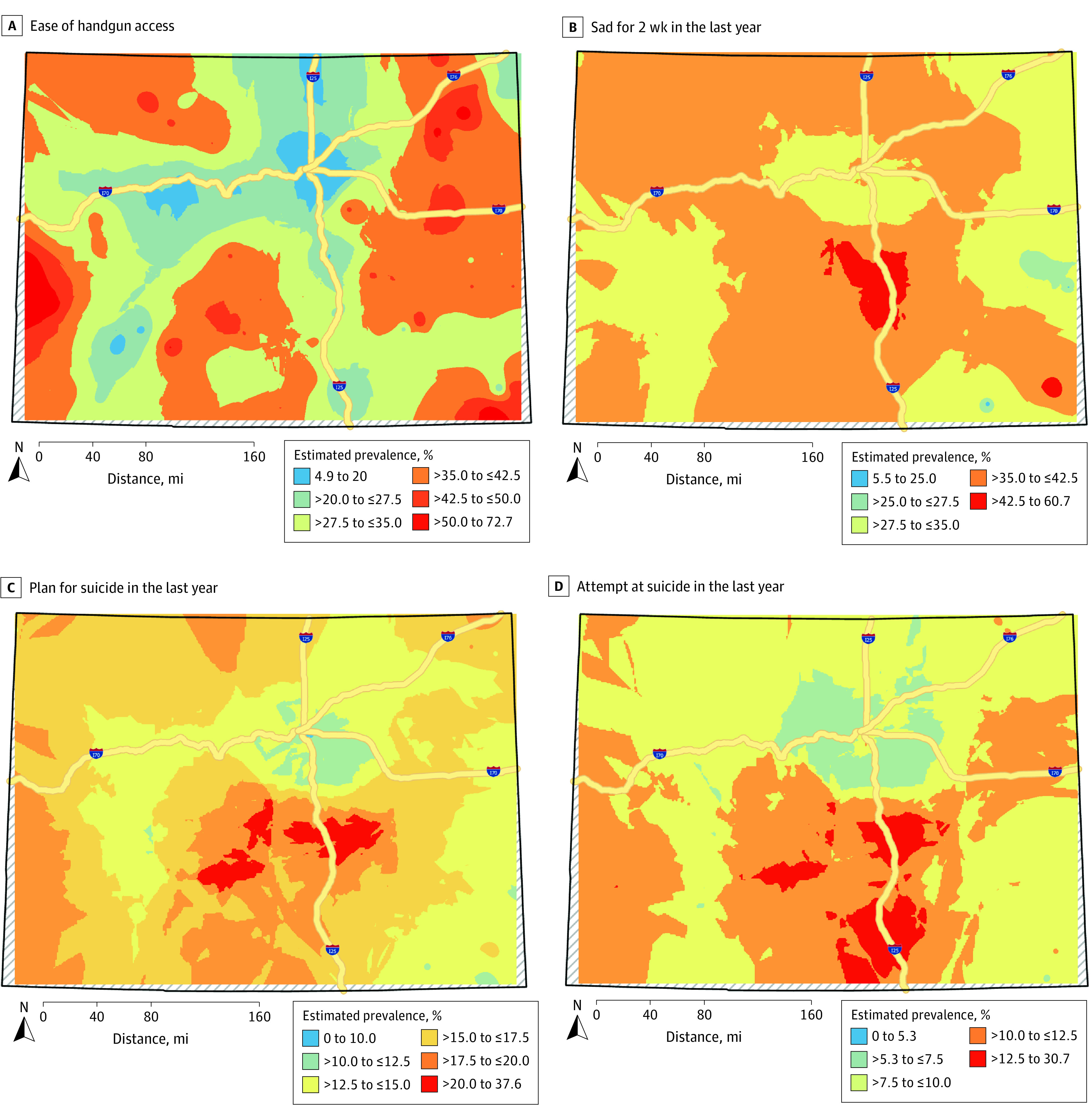
Smooth-Surface Estimates for Weighted Prevalence of Survey Responses Surface smoothed using empirical Bayesian kriging in ArcGIS Pro version 4.6.0. Estimated prevalence could only be interpolated prevalence within the boundaries of the farthest Colorado schools, thus the surface ends short of the state line. Different intervals for each measure were created to highlight areas with relative higher and lower prevalence.

Maps visualizing smoothed weighted prevalence for handgun access (Figure 2A) show higher prevalence in both the eastern plains and the mountainous western areas and lower prevalence along the Interstate-25 corridor, where 85% of the Colorado population lives.^35^ Suicidality measures (excluding considering suicide because it was not spatially dependent) were highest in the south central region of the state, with higher prevalence areas along the western slope of the Rocky Mountains (Figure 2B-D).

Local Moran *I* results showed a similar spatial pattern to the maps presented in Figure 2. Because of this and confidentiality concerns, we chose not to present separate local Moran *I* maps but identified 27 schools within significant hot spots with easy handgun access, meaning they, along with neighboring schools, had high prevalence of easy handgun access. Of these, 19 schools (70.4%) were rural (remote) and 8 schools (29.6%) were rural (distant) or town (distant/remote). We identified 78 schools as significant cold spots (37 schools (47.4%) classified as city [large] or city [midsize/small]), meaning that they had low prevalence of easy gun access and were located near other low prevalence schools. There were 9 schools (2 of which were rural), mostly in south central Colorado, that were identified as hot spots for both prevalence of planning suicide and attempting suicide in the last year. Cold spots for these variables were mostly concentrated around the Denver metropolitan area. There were no hot spots for easy handgun access and suicidality, while cold spots for both overlapped in the Denver metropolitan area. Sensitivity analysis indicated no meaningful difference in findings when using a different weight matrix.

**Figure 3.**
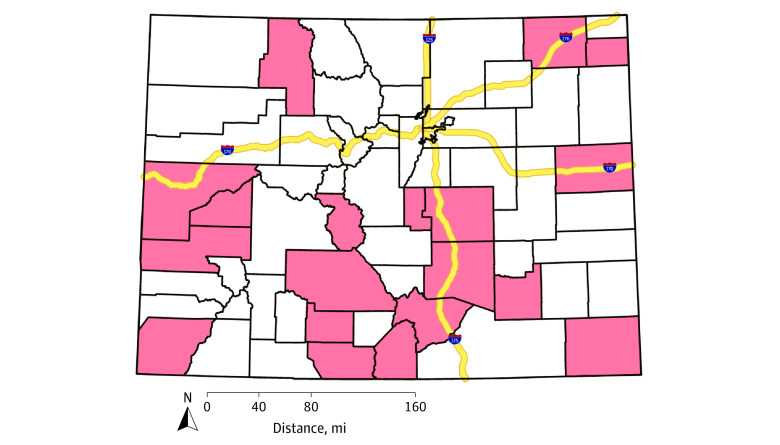
Counties With at Least 1 School Identified as Potentially at High Risk of Youth Suicides Schools meeting this definition had both prevalence of easy handgun access and prevalence of students planning a suicide attempt in the last year in the top quartile. High-risk counties are pink.

Finally, we identified schools with high weighted prevalence of easy handgun access and planning suicide, identifying 21 schools in 19 counties with a prevalence in the top quartile for both measures (Figure 3). Of these, 13 schools (61.9%) were rural (remote), 4 schools (19.0%) were rural (distant), and 4 schools (19.0%) were town (distant/remote).

## Discussion

This cross-sectional study explored school-level prevalence of self-reported ease of access to handguns and suicidality in Colorado youth, analyzing these associations for spatial clustering and by rurality. Consistent with previous findings,^36,37,38^ we found that access to firearms increased with increasing rurality. Previous research has reported that firearms are the most lethal method for suicide attempts^13,14^ and increased access to firearms is associated with higher suicide fatality rates.^4,5,6,7,8,9,10^ Schools in the most remote and rural areas had the highest prevalence of reported easy handgun access. We also identified 19 counties with at least 1 school with highest prevalence of both easy handgun access and planning suicide in the previous year.

### Rurality

We found that rurality was associated with prevalence of easy handgun access but not necessarily with measures of suicidality. Rurality of school was most strongly correlated with students reporting easy handgun access, with more than one-third of students in rural (remote) schools reporting easy access, 2-fold higher than their city (large) counterparts. Because the survey asked youth about their perceived access to handguns, it is unclear whether we captured differences in actual access to handguns. For example, youth could answer yes if there is an unlocked or otherwise accessible handgun in their house, a family member or friend’s house, or they felt they could easily purchase one. The multiple potential reasons for this response would require different community-level interventions. However, since these findings are consistent with previous research into rural-urban differences in firearm ownership,^20^ this question is likely capturing an important phenomenon.

Some measures of suicidality were highest in rural (remote) and rural (distant) schools, although not in rural (fringe) schools, indicating some, but not all, rural students reported struggles. Research in adults has found increasing suicidality in rural areas, with many possible causes, such as social isolation and low resources (eg, mental health services).^17,39,40,41^ The association between rurality and suicidality in youth needs further investigation.

### Geography

While rurality was strongly correlated with handgun access, looking beyond urban-rural differences and mapping measures at the substate-level provided rich information to inform public health action. To our knowledge, this study is the first to explore the spatial distribution of youth handgun access. Easy handgun access showed more spatial clustering than did suicidality measures, with hot spots of high handgun access around the periphery of the state, predominantly in rural areas. Combining empirical Bayesian kriging with local Moran *I* allowed us to visualize and identify areas with especially high prevalence of reported easy handgun access.

We did not observe consistent spatial overlap in easy handgun access and suicidality hot spots statewide. However, we identified counties with at least 1 school with both higher prevalence of self-reported easy handgun access and suicidality. Previous research has reported that when a person is suicidal, having easy access to a firearm can be lethal.^11,24,42^ Therefore, our study contributes to the identification of schools and communities with potential for higher ease of access to firearms when youth are at risk of suicide.

### Implications

The social ecological model promotes looking beyond individual-level interventions to the community or societal level to achieve population-level impact.^43^ Using school-level prevalence allowed for identification of communities potentially at highest risk of firearm suicides. These findings draw attention to schools or communities that might benefit most from population-based suicide interventions. Selecting and targeting localities at highest risk could have a bigger impact on health outcomes,^44^ allowing for directed and tailored firearm suicide interventions to improve the effectiveness and use of limited resources in adolescent suicide prevention efforts.

While understanding rural-urban differences is important for geographically appropriate approaches to prevention, not all rural areas have the same risk. This study demonstrated that using school-level data could better specify where risk of firearm access and suicide were higher.

### Limitations

This study has several limitations. First, data were self-reported by high school students, although research indicates that overall, high school students accurately respond to confidential surveys.^25^ Second, the survey question specifically asks about access to handguns. In rural counties, long guns are used in half of adolescent suicides, although other studies have reported that handgun use in suicides is increasingly common.^37,45^ The impact of the wording of the question on findings is not entirely clear; the 2015 National Firearms Survey found that gun owners had a mean of 4.8 guns^20^ and 75% of gun owners with children in the home owned at least 1 handgun.^15^ Therefore, it is possible that the results would not differ drastically if the survey question asked about easy access to any firearm type. Additionally, not all Colorado high schools participated in HKCS. Although we identified schools and communities that would likely benefit most from intervention, there could be other schools and regions that also have high measures of handgun access and suicidality that were not surveyed.

Comparison of our findings with other studies is difficult because of both lack of national surveillance data on firearm access and suicide attempts and low base rates of youth suicide mortality, especially at smaller geographies. Therefore, this study provides an important contribution to understanding youth firearm access and suicidality using school-level data to indicate possible school and community risk of youth firearm suicide. Future studies should track firearm access and suicidality measures over time and explore how contextual information may help explain trends. Additionally, measures of firearm access regarding handguns and long guns should be included in other national surveillance systems (eg, Youth Risk Behavioral Surveillance System and Behavioral Risk Factor Surveillance System) to explore patterns and identify higher-risk areas in other states.

## Conclusions

This cross-sectional study identified schools and communities with high prevalence of suicidality and handgun access. In Colorado, rural and remote communities with high prevalence of easy handgun access and suicidality may benefit most from reducing access to lethal means when a young person is in crisis. Ongoing investment in interventions across the socioecological model to temporarily reduce youth access to firearms when youth are in crisis is a necessary next step for preventing youth suicides. National surveillance systems should consider including questions about firearm ownership and access to improve research and public health action to prevent firearm-related mortality.

## Notes

Supplement. **eTable 1.** Urban-Centric Locale Definitions **eTable 2.** Demographic and Geographic Weighted Prevalence of the Sample

## References

[1] Heron M. Deaths: leading causes for 2017. Natl Vital Stat Rep. 2019;68(6):1-77. 32501203

[2] Curtin SC. State suicide rates among adolescents and young adults aged 10-24: United States, 2000-2018. Natl Vital Stat Rep. 2020;69(11):1-10. 33054915

[3] Centers for Disease Control and Prevention. WISQARS—Web-based Injury Statistics Query and Reporting System. Accessed January 4, 2018. https://www.cdc.gov/injury/wisqars

[4] Miller M, Warren M, Hemenway D, Azrael D. Firearms and suicide in US cities. Inj Prev. 2015;21(e1):e116-e119. doi:10.1136/injuryprev-2013-040969 24302479

[5] Johnson RM, Barber C, Azrael D, Clark DE, Hemenway D. Who are the owners of firearms used in adolescent suicides? Suicide Life Threat Behav. 2010;40(6):609-611. doi:10.1521/suli.2010.40.6.609 21198329 PMC3085447

[6] Knopov A, Sherman RJ, Raifman JR, Larson E, Siegel MB. Household gun ownership and youth suicide rates at the state level, 2005-2015. Am J Prev Med. 2019;56(3):335-342. doi:10.1016/j.amepre.2018.10.027 30661885 PMC6380939

[7] Grossman DC, Reay DT, Baker SA. Self-inflicted and unintentional firearm injuries among children and adolescents: the source of the firearm. Arch Pediatr Adolesc Med. 1999;153(8):875-878. doi:10.1001/archpedi.153.8.875 10437764

[8] Anglemyer A, Horvath T, Rutherford G. The accessibility of firearms and risk for suicide and homicide victimization among household members: a systematic review and meta-analysis. Ann Intern Med. 2014;160(2):101-110. doi:10.7326/M13-1301 24592495

[9] Kellermann AL, Rivara FP, Somes G, . Suicide in the home in relation to gun ownership. N Engl J Med. 1992;327(7):467-472. doi:10.1056/NEJM199208133270705 1308093

[10] Brent DA, Perper JA, Allman CJ, Moritz GM, Wartella ME, Zelenak JP. The presence and accessibility of firearms in the homes of adolescent suicides: a case-control study. JAMA. 1991;266(21):2989-2995. doi:10.1001/jama.1991.03470210057032 1820470

[11] Betz ME, Barber C, Miller M. Suicidal behavior and firearm access: results from the second injury control and risk survey. Suicide Life Threat Behav. 2011;41(4):384-391. doi:10.1111/j.1943-278X.2011.00036.x 21535097

[12] Miller M, Barber C, Azrael D, Hemenway D, Molnar BE. Recent psychopathology, suicidal thoughts and suicide attempts in households with and without firearms: findings from the National Comorbidity Study Replication. Inj Prev. 2009;15(3):183-187. doi:10.1136/ip.2008.021352 19494098

[13] Miller M, Azrael D, Hemenway D. The epidemiology of case fatality rates for suicide in the northeast. Ann Emerg Med. 2004;43(6):723-730. doi:10.1016/j.annemergmed.2004.01.018 15159703

[14] Vyrostek SB, Annest JL, Ryan GW. Surveillance for fatal and nonfatal injuries—United States, 2001. MMWR Surveill Summ. 2004;53(7):1-57. 15343143

[15] Azrael D, Cohen J, Salhi C, Miller M. Firearm storage in gun-owning households with children: results of a 2015 national survey. J Urban Health. 2018;95(3):295-304. doi:10.1007/s11524-018-0261-7 29748766 PMC5993703

[16] Branas CC, Nance ML, Elliott MR, Richmond TS, Schwab CW. Urban-rural shifts in intentional firearm death: different causes, same results. Am J Public Health. 2004;94(10):1750-1755. doi:10.2105/AJPH.94.10.1750 15451745 PMC1448529

[17] Hirsch JK, Cukrowicz KC. Suicide in rural areas: an updated review of the literature. J Rural Ment Heal. 2014;38(2):65-78. doi:10.1037/rmh0000018

[18] Choi NG, DiNitto DM, Nathan Marti C. Differences in firearm suicides by residential location in Texas, 2006-2015. Arch Suicide Res. 2019;23(3):491-506. doi:10.1080/13811118.2018.1468290 29791268

[19] Fontanella CA, Hiance-Steelesmith DL, Phillips GS, . Widening rural-urban disparities in youth suicides, United States, 1996-2010. JAMA Pediatr. 2015;169(5):466-473. doi:10.1001/jamapediatrics.2014.3561 25751611 PMC4551430

[20] Azrael D, Hepburn L, Hemenway D, Miller M. The stock and flow of U.S. firearms: results from the 2015 National Firearms Survey. RSF Russell Sage Found J Soc Sci. 2017;3(5):38-57. doi:10.7758/rsf.2017.3.5.02

[21] Siegel M, Ross CS, King C III. A new proxy measure for state-level gun ownership in studies of firearm injury prevention. Inj Prev. 2014;20(3):204-207. doi:10.1136/injuryprev-2013-040853 23956369

[22] Schell T, Peterson S, Vegetabile B, Scherling A, Smart R, Morral A. State-Level Estimates of Household Firearm Ownership. RAND Corporation; 2020. doi:10.7249/tl354

[23] Brooks-Russell A, Ma M, Brummett S, Wright-Kelly E, Betz M. Perceived access to handguns among Colorado high school students. Pediatrics. 2021;147(4):e2020015834. doi:10.1542/peds.2020-015834 33782105

[24] Florentine JB, Crane C. Suicide prevention by limiting access to methods: a review of theory and practice. Soc Sci Med. 2010;70(10):1626-1632. doi:10.1016/j.socscimed.2010.01.029 20207465

[25] Brener ND, Kann L, Shanklin S, ; Centers for Disease Control and Prevention. Methodology of the youth risk behavior surveillance system—2013. MMWR Recomm Rep. 2013;62(RR-1):1-20. 23446553

[26] Communities that Care. Risk and protective factor scale construction summary. Accessed April 27, 2021. http://healthyyouth.org/documents/Riskprotfactorconstructionsummary.pdf

[27] Geverdt D; National Center for Education Statistics. Education Demographic and Geographic Estimates (EDGE) Program: locale boundaries user’s manual. Accessed April 27, 2021. https://nces.ed.gov/programs/edge/docs/EDGE_NCES_LOCALE.pdf

[28] Colorado Department of Public Health and Environment Open Data. CDPHE CDOE school locations and district office locations. Accessed April 27, 2021. https://data-cdphe.opendata.arcgis.com/datasets/fec1a4755e7f454389dcd18e183c8e08_0

[29] Rosner B. Fundamentals of Biostatistics. 7th ed. Duxbury; 2006.

[30] Brownstein JS, Cassa CA, Mandl KD. No place to hide—reverse identification of patients from published maps. N Engl J Med. 2006;355(16):1741-1742. doi:10.1056/NEJMc061891 17050904

[31] Cliff A, Ord J. Spatial Process—Models & Applications. Pion; 1981.

[32] Anselin L, Morrison G. Distance-based spatial weights. Accessed April 27, 2021. https://spatialanalysis.github.io/lab_tutorials/Distance_Based_Spatial_Weights.html#distance-band-weights

[33] Anselin L. Local indicators of spatial organization—LISA. Geogr Anal. 1995;27(2):93-115. doi:10.1111/j.1538-4632.1995.tb00338.x

[34] Krivoruchko K. Empirical Bayesian kriging: implemented in ArcGIS geostatistical analyst. Accessed April 27, 2021. https://www.esri.com/NEWS/ARCUSER/1012/files/ebk.pdf

[35] Colorado Department of Local Affairs. Population totals for Colorado counties. Accessed April 27, 2021. https://demography.dola.colorado.gov/population/population-totals-counties/#population-totals-for-colorado-counties

[36] Swanson SA, Eyllon M, Sheu Y-H, Miller M. Firearm access and adolescent suicide risk: toward a clearer understanding of effect size. Inj Prev. 2020;7(3):264-270. doi:10.1136/injuryprev-2019-043605 32409621 PMC8165151

[37] Hanlon TJ, Barber C, Azrael D, Miller M. Type of firearm used in suicides: findings from 13 states in the National Violent Death Reporting System, 2005-2015. J Adolesc Health. 2019;65(3):366-370. doi:10.1016/j.jadohealth.2019.03.015 31227389

[38] Simonetti JA, Mackelprang JL, Rowhani-Rahbar A, Zatzick D, Rivara FP. Psychiatric comorbidity, suicidality, and in-home firearm access among a nationally representative sample of adolescents. JAMA Psychiatry. 2015;72(2):152-159. doi:10.1001/jamapsychiatry.2014.1760 25548879

[39] Fontanella CA, Saman DM, Campo JV, . Mapping suicide mortality in Ohio: a spatial epidemiological analysis of suicide clusters and area level correlates. Prev Med. 2018;106:177-184. doi:10.1016/j.ypmed.2017.10.033 29133266

[40] Steelesmith DL, Fontanella CA, Campo JV, Bridge JA, Warren KL, Root ED. Contextual factors associated with county-level suicide rates in the United States, 1999 to 2016. JAMA Netw Open. 2019;2(9):e1910936. doi:10.1001/jamanetworkopen.2019.10936 31490540 PMC6735416

[41] Dresang LT. Gun deaths in rural and urban settings: recommendations for prevention. J Am Board Fam Pract. 2001;14(2):107-115. 11314917

[42] Dempsey CL, Benedek DM, Zuromski KL, . Association of firearm ownership, use, accessibility, and storage practices with suicide risk among US Army soldiers. JAMA Netw Open. 2019;2(6):e195383. doi:10.1001/jamanetworkopen.2019.5383 31173124 PMC6563574

[43] Allchin A, Chaplin V, Horwitz J. Limiting access to lethal means: applying the social ecological model for firearm suicide prevention. Inj Prev. 2019;25(suppl 1):i44-i48. doi:10.1136/injuryprev-2018-042809 29941633

[44] Keyes KM, Galea S. Population Health Science. Oxford University Press; 2016. doi:10.1093/med/9780190459376.001.0001

[45] Nestadt PS, MacKrell K, McCourt AD, Fowler DR, Crifasi CK. Prevalence of long gun use in Maryland firearm suicides. Inj Epidemiol. 2020;7(1):4. doi:10.1186/s40621-019-0230-y 32127045 PMC6996182

