# Supplementary material for: Assessment of Rural-Urban and Geospatial Differences in Perceived Handgun Access and Reported Suicidality Among Youth in Colorado
Source: JAMA Netw Open. 2021 Oct 8;4(10):e2127816. doi: 10.1001/jamanetworkopen.2021.27816 (PMC8501400; doi:10.1001/jamanetworkopen.2021.27816)
Supplement: Supplement. — eTable 1. Urban-Centric Locale Definitions eTable 2. Demographic and Geographic Weighted Prevalence of the Sample [file jamanetwopen-e2127816-s001.pdf]

Supplemental Online Content

Spark TL, Wright-Kelly E, Ma M, James KA, Reid CE, Brooks-Russell A. Assessment of rural-urban and geospatial differences in perceived handgun access and reported suicidality among youth in Colorado. *JAMA Netw Open*. 2021;4(10):e2127816. doi:10.1001/jamanetworkopen.2021.27816

eTable 1. Urban Centric Locale Definitions
eTable 2. Demographic and Geographic Weighted Prevalence of the Sample

This supplemental material has been provided by the authors to give readers additional information about their work.

© 2021 Spark TL et al. *JAMA Network Open.*

eTable 1. Urban Centric Locale Definitions[a]

Urban Centric Locale	Definition
City Large	Territory inside an Urbanized Area and inside a Principal City with population of 250,000 or more
City Midsize	Territory inside an Urbanized Area and inside a Principal City with population less than 250,000 and greater than or equal to 100,000.
City Small	Territory inside an Urbanized Area and inside a Principal City with population less than 100,000.
Suburban Large	Territory outside a Principal City and inside an Urbanized Area with population of 250,000 or more.
Suburban Midsize	Territory outside a Principal City and inside an Urbanized Area with population less than 250,000 and greater than or equal to 100,000.
Suburban Small	Territory outside a Principal City and inside an Urbanized Area with population less than 100,000.
Town Fringe	Territory inside an Urban Cluster that is less than or equal to 10 miles from an Urbanized Area.
Town Distant	Territory inside an Urban Cluster that is more than 10 miles and less than or equal to 35 miles from an Urbanized Area.
Town Remote	Territory inside an Urban Cluster that is more than 35 miles from an Urbanized Area.
Rural Fringe	Census-defined rural territory that is less than or equal to 5 miles from an Urbanized Area, as well as rural territory that is less than or equal to 2.5 miles from an Urban Cluster.
Rural Distant	Census-defined rural territory that is more than 5 miles but less than or equal to 25 miles from an Urbanized Area, as well as rural territory that is more than 2.5 miles but less than or equal to 10 miles from an Urban Cluster.
Rural Remote	Census-defined rural territory that is more than 25 miles from an Urbanized Area and also more than 10 miles from an Urban Cluster.

[a] Urban Centric Locale Designation provided by National Center for Education Statistics defined based on proximity to principal cities and urbanized areas. More information can be found at: https://nces.ed.gov/programs/edge/docs/EDGE_NCES_LOCALE.pdf

eTable 2. Demographic and Geographic Weighted Prevalence of the Sample

		Urban Centric Locale[a]				
	**Overall**	**City**	**Suburb**	**Town**	**Rural**	***p***
**Number of Schools**<br>**Number of Students**	258<br>59,602	59<br>17,801	52<br>16,605	43<br>12,112	104<br>13,084	
**Weighted Number of Students**	187,288	69,328	71,467	20,608	25,885	
**Gender**						0.34
**Male**	49.7<br>(49.3, 50.1)	49.8<br>(49.1, 50.5)	50.3<br>(49.5, 51.0)	50.7<br>(49.8, 51.6)	50.7<br>(49.9, 51.6)	
**Female**	50.3<br>(49.9, 50.7)	50.2<br>(49.5, 50.9)	49.7<br>(49.0, 50.5)	49.3<br>(48.4, 50.2)	49.3<br>(48.4, 50.1)	
**Grade**						0.008
**9th**	27.0<br>(26.6, 27.4)	26.9<br>(26.2, 27.5)	26.5<br>(25.8, 27.2)	28.0<br>(27.2, 28.8)	26.9<br>(26.2, 27.7)	
**10th**	26.9<br>(26.6, 27.3)	26.9<br>(26.2, 27.5)	27.5<br>(26.8, 28.2)	27.0<br>(26.2, 27.8)	26.2<br>(25.5, 27.0)	
**11th**	25.1<br>(24.7, 25.4)	24.6<br>(23.9, 25.2)	25.3<br>(24.6, 26.0)	24.9<br>(24.1, 25.6)	25.6<br>(24.9, 26.4)	
**12th**	21.0<br>(20.7, 21.4)	21.7<br>(21.1, 22.3)	20.8<br>(20.1, 21.4)	20.2<br>(19.5, 20.9)	21.2<br>(20.5, 21.9)	
**Race/Ethnicity**						<.0001
**Non-Hispanic White**	50.8<br>(50.4, 51.2)	36.5<br>(35.7, 37.2)	59.6<br>(58.8, 60.4)	50.8<br>(50.0, 51.7)	59.3<br>(58.5, 60.2)	
**Non-Hispanic Black**	3.4<br>(3.3, 3.6)	7.6<br>(7.2, 8.0)	2.6<br>(2.3, 2.8)	1.1<br>(1.0, 1.3)	1.0<br>(0.8, 1.1)	
**Hispanic**	36.4<br>(36.0, 36.8)	44.6<br>(43.9, 45.3)	27.1<br>(26.4, 27.8)	42.2<br>(41.3, 43.1)	31.6<br>(30.8, 32.4)	
**Other**	9.4<br>(9.1, 9.6)	11.4<br>(10.9, 11.8)	10.7<br>(10.3, 11.2)	5.8<br>(5.4, 6.2)	8.1<br>(7.7, 8.6)	
**Sexual Orientation**						<.0001
**Heterosexual**	87.7<br>(87.4, 88.0)	85.6<br>(85.1, 86.2)	87.5<br>(87.0, 88.1)	89.2<br>(88.7, 89.8)	89.4<br>(88.8, 89.9)	
**Gay/lesbian/bi-sexual**	12.3<br>(12.0, 12.6)	14.4<br>(13.8, 14.9)	12.5<br>(11.9, 13.0)	10.8<br>(10.2, 11.3)	10.6<br>(10.1, 11.2)	
**Gender Identity**						0.02
**Cisgender**	98.6<br>(98.5, 98.7)	98.6<br>(98.4, 98.8)	98.3<br>(98.1, 98.5)	98.7<br>(98.5, 98.9)	98.8<br>(98.6, 99.0)	
**Transgender**	1.4<br>(1.3, 1.5)	1.4<br>(1.2, 1.6)	1.7<br>(1.5, 1.9)	1.3<br>(1.1, 1.5)	1.2<br>(1.0, 1.4)	
**Mother's Education**						<.0001
**High school or less**	36.4<br>(36.0, 36.8)	40.3<br>(39.5, 41.1)	29.1<br>(28.3, 29.8)	41.1<br>(40.2, 42.1)	36.4<br>(35.6, 37.3)	
**Some college**	15.2<br>(14.9, 15.5)	14.9<br>(14.3, 15.4)	15.3<br>(14.7, 15.9)	14.6<br>(14.0, 15.3)	16.1<br>(15.4, 16.8)	
**College or grad school**	48.4<br>(47.9, 48.8)	44.9<br>(44.1, 45.7)	55.6<br>(54.8, 56.5)	44.2<br>(43.2, 45.2)	47.5<br>(46.5, 48.4)	

[a] Urban Centric Locale Designation provided by National Center for Education Statistics defined based on proximity to principal cities and urbanized areas. The twelve categories were collapsed into four larger groups.

© 2021 Spark TL et al. *JAMA Network Open.*
